# Standing on the Shoulders of Giant Viruses: Five Lessons Learned about Large Viruses Infecting Small Eukaryotes and the Opportunities They Create

**DOI:** 10.1371/journal.ppat.1005752

**Published:** 2016-08-25

**Authors:** Steven W. Wilhelm, Samantha R. Coy, Eric R. Gann, Mohammad Moniruzzaman, Joshua M. A. Stough

**Affiliations:** Department of Microbiology, The University of Tennessee, Knoxville, Tennessee, United States of America; University of Florida, UNITED STATES

## Introduction

Viruses are generally considered to be amongst the smallest bioactive particles; dating back to the original observations, including those of luminaries such as Ivanosky and Beijerinck, size has always been at issue within the definition, a tradition that continued for many years [1]. It was thus a surprise to the scientific community in the early 2000s when French scientists demonstrated that a particle, previously thought to be a bacterium, was indeed a virus [2]. The discovery of the Mimivirus and the other “giants” that have followed, including Mamavirus, Pandoravirus, Faustovirus, and Mollivirus, has blurred the definition of what constitutes a virus and, indeed, the boundaries between viral particles and cellular life [3].

## What Are “Giant Viruses”?

In general, the term “giant virus” is now commonly used to refer to viruses that have a large genome (>200,000 base pairs) and/or particle size (>0.2 μm). While a variety of arguments can be made for altering these metrics, what is clear is that these viruses bring with them a potential (in terms of genes that are transcribed and translated) that is historically associated with cellular life forms: this includes members of the *Mimiviridae* that infect amoebas, as well as the “extended” phylogenetic group that infect algae [4]. These viruses fall into the Nucleocytoplasmic large DNA viruses (NCLDVs) group that also includes the *Phycodnaviridae*, *Iridoviridae*, *Poxviridae*, *Marseilleviridae*, *Asfarviridae*, and *Ascoviridae*. These viruses have been shown (to date) to infect organisms including algae and protists (although members of *Poxviridae*, *Asfarviridae*, and *Iridoviridae* infect humans and animals). And while their size is constantly surprising, it is the latter trait (i.e., their novel genetic collection) that is of interest to many researchers. A research opportunity is to question why these particles need to carry so many different genetic blueprints (i.e., genes), and how these extra costs provide benefits with respect to viral fitness and selection in a complicated and complex microbial and viral world.

## Have We Really Only Known about Giant Viruses for Little More Than a Decade?

As noted above, the study of giant viruses emerged with the confluence of observation (the ability to see Mimivirus in a light microscope and the realization that it was a virus and not a bacterium) and opportunity (the emergence of modern techniques in molecular biology that have rapidly advanced the ability to study these viruses). In retrospect, there have historically been many other large viruses observed by scientists. Several algal viruses such as Ectocaprus siliculosus virus (EsV-1) [5] and Micromonas pusilla virus (MpV) [6] were observed decades ago and have been studied sporadically over the years. Others, highlighted by Emiliania huxleyi virus (EhV) [7] and perhaps the best-studied of the large virus systems, the *Chlorella*-infecting virus group [8], have been characterized in studies ranging from biochemical to emerging ecological studies in recent years. But a survey of the literature reveals many more opportunities for research with completely new giant virus–host systems. From the early 1970s through the 1990s, more broadly available imaging tools allowed researchers to observe giant virus-like particles in hosts that have yet to be deeply studied, including 240- and 390-nm particles found in the chlorophytes *Oedogonium* spp. “L” [9] and *Uronema gigas* [10], respectively, as well as a 385-nm virus particle in the dinoflagellate *Gymnodinium uberrimum* [11]. Indeed, an opportunity exists for researchers to take advantage of the availability of this information to identify new virus–host models for laboratory study. As is clear from the foundational work in Chloroviruses and Mimiviruses, there are rich prospects to advance science through the thorough isolation and study of new virus–host systems.

## How Broadly Are Giant Viruses Distributed in Nature?

**Fig 1:**
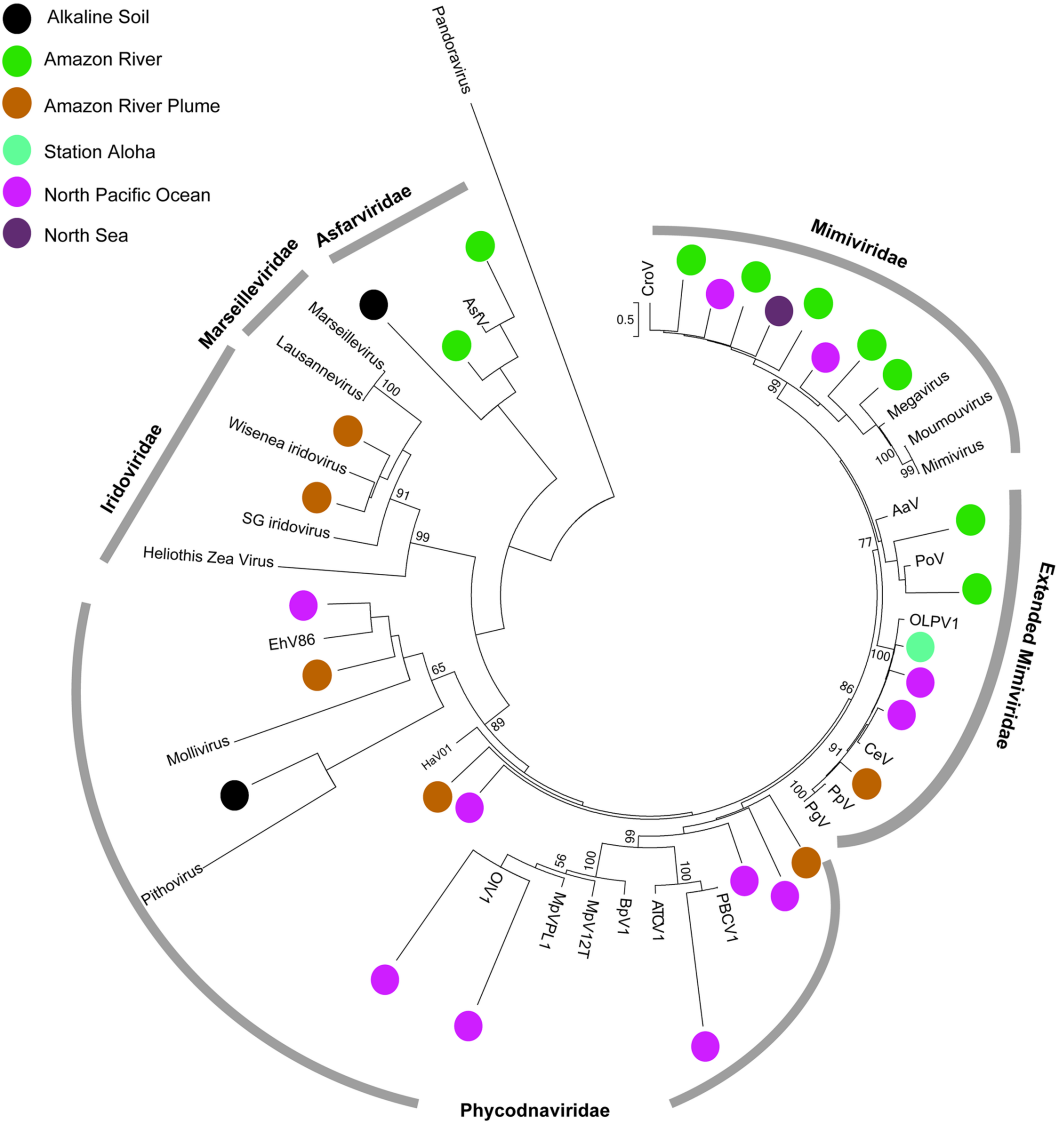
Phylogenetic reconstruction of NCLDV major capsid protein sequences from environmental metatranscriptomes generated from an alkaline soil sample (NCBI ID: SRP043976), the Amazon River and River Delta (SRP037995, SRP039544 [21]), the North Pacific Ocean (SRP052554 [22]), Station ALOHA in the tropical Pacific Ocean (CAM_SMPL_000824 at iMicrobe.us [23]), and the North Sea (ERP004582 [24]). Public metatranscriptomes were assembled and searched for NCLDV-like major capsid protein coding transcripts. MCP contigs >300 bp with best hits to NCLDVs were aligned with an MCP reference database and placed on a maximum likelihood tree with a Shimodaira-Hasegawa-like approximate likelihood ratio test branch validation using *pplacer* (http://matsen.fhcrc.org/pplacer/). The broad spectrum of samples demonstrates active giant virus infections can be observed in many different environments.

While the discovery of Mimivirus has spurred extensive research into the origins and capabilities of giant viruses, little is currently known about their global distribution and diversity. Giant viruses have been isolated largely from aquatic samples, often using *Acanthamoeba* spp. to enrich for virus populations. This technique has led to the discovery of novel giant viruses in marine and freshwater samples and, unexpectedly, ancient amoeba-infecting viruses that have persisted in ~30,000-year-old Siberian permafrost [12]. Giant viruses have even recently been isolated from humans and may be linked to various disease/disorder states [13]. That the discovery of so many new giant viruses is surprising suggests that classical methods need to be reconsidered for the study of giant viruses. One tool that may help to address the question of giant virus diversity is the analysis of molecular sequencing data. Researchers have already found markers for giant viruses and, using shotgun and targeted metagenomes, have shown these particles to be distributed across a broad spectrum of environments (e.g., [14]). Indeed, publicly available meta-genomic/transcriptomic sequences from environmental samples may serve the same purpose as the archived images of virus infection processes. An emergent opportunity is that existing knowledge of giant virus genomic sequences, such as the conserved major capsid protein (MCP) (Fig 1), can be used to probe the wealth of available sequencing data. In addition, transcriptomics (i.e., RNA sequencing) data for these giant viruses may provide information on active infections, again providing opportunities to identify specific virus–host relationships in future work. As we move forward in this arena, a second opportunity, the development of in situ approaches to study giant virus impacts on ecosystem scale ecology, also emerges.

## What Can Giant Viruses Do That Makes Them Special?

Giant viruses exhibit diverse morphologies, lifestyles, and even genomic structure, but there are some shared features that set them apart from other systems. Most obvious is the “nucleocytoplasmic” distinction of replicative strategies: for example, in some of these giant viruses, a cytoplasmic, organelle-like virion factory quickly forms in the infected host as the site for virion morphogenesis—a feature previously witnessed only in RNA viruses [15]. This organization is thought to optimize control of intracellular resources and may be particularly important where viral infection has been shown to alter metabolic processes. Another hallmark of giant viruses is the aforementioned gene content previously observed only in cellular organisms. Indeed, this includes (but is not limited to) central components of protein translation, parts of DNA repair pathways, polysaccharide synthesis enzymes, genes containing inteins, and, more recently, evidence for a genetic system that may offer protection against virion factory-infecting virophage [16]. These features indicate that giant viruses, unlike smaller lytic phages, encode for much more than the blueprints for generating new viruses. Interestingly, the location of these elements on the viral genome appears to influence gene conservation. For example, after being subcultured for several generations in a germ-free amoebal host, Mimivirus experienced a 17% genome reduction, with most gene losses occurring at the terminal ends [17]. Aureococcus anophagefferens virus (AaV) may also use this mechanism, as recent studies indicate that horizontally acquired genes often occur in the terminal regions [4]. Indeed, an emergent opportunity is to characterize the mechanics of how these viruses gain and lose genetic information: for example, it would be interesting to see if the “genetic accordion” theory [18] is at play in other large viruses, and if the mechanisms involved can result in not just duplications but in the horizontal transfer of materials from the hosts to viruses.

## How Do Studies of Giant Viruses Shape Scientific Knowledge in a Larger Context?

One thing that has become clear since the discovery of the Mimivirus (and reinforced by the subsequent isolation of Pandoravirus, Pithovirus, and others) is that the “rules” are changing. In recent months, we have seen a series of researchers working to redefine “What is a virus?” [19] and even “What is life?” [20]. Apparent, but not always on the forefront of this debate, is how these viruses have changed life as we understand it. This statement is not to open the debate on what is life, but on how life has evolved and continues to evolve in the presence of these viruses. Within the genomes of these so-called giant viruses are genes that have been attributed to a variety of lineages: indeed, in just one example (AaV), we find a collection of genes that are phylogenetically most closely related to other giant viruses, to the host, to other picoeukaryotes, to bacteria and archaea, and even to phage [4]. The presence of these viruses and their often unique genomic architecture suggest that the horizontal transfer of genes between viruses and hosts as well as from organisms consumed by hosts (e.g., during phagotrophy) to viruses is likely rampant. These observations and the availability of a growing number of virus–host systems create an opportunity to study these dynamics in the laboratory. Just as researchers have now established long-term evolution experiments with microbes, there is an opportunity to follow the long-term evolution of these virus–host systems.

## References

[1] Lwoff A. The concept of a virus. Journal of General Microbiology. 1957;17(2):239–53. PubMed Central PMCID: PMC13481308. 13481308 10.1099/00221287-17-2-239

[2] La Scola B, Audic S, Robert C, Jungang L, de Lamballerie X, Drancourt M, et al A giant virus in amoebae. Science. 2003;299:2033 10.1126/science.1081867. PubMed Central PMCID: PMC12663918. 12663918

[3] Abergel C, Legendre M, Claverie JM. The rapidly expanding universe of giant viruses: Mimivirus, Pandoravirus, Pithovirus and Mollivirus. FEMS Microbiol Rev. 2015;39(6):779–96. 10.1093/femsre/fuv037 . 26391910

[4] Moniruzzaman M, LeCleir GR, Brown CM, Gobler CJ, Bidle KD, Wilson WH, et al Genome of the Brown Tide virus (AaV), the little giant of the Megaviridae, elucidates NCLDV genome expansion and host-virus coevolution. Virology. 2014;466–467:60–70. 10.1016/j.virol.2014.06.031 25035289

[5] Muller DG, Kawai H, Stache B, Lanka S. A virus infection in the marine brown alga *Ectocarpus siliculosus* (Phaeophyceae). Botanica Acta. 1990;103:72–82.

[6] Mayer JA, Taylor FJR. A virus which lyses the marine nanoflagellate *Micromonas pusilla* . Nature. 1979;281:299–301.

[7] Bratbak G, Wilson W, Heldal M. Viral control of *Emiliania huxleyi* blooms. Journal of Marine Systems. 1995;9:75–81.

[8] Van Etten JL, Meints RH, Kuczmarski D, Burbank DE, Lee K. Viruses of symbiotic Chlorella-like algae isolated from *Paramecium bursana* and *Hydra viridis* . PNAS. 1982;79:3867–71. 16593198 10.1073/pnas.79.12.3867 PMC346529

[9] Pickett-Heaps JD. A possible virus infection in the green alga *Oedogonium* . J Phycol. 1972;8:44–7.

[10] Dodds JA, Cole A. Microscopy and biology of *Uronema gigas*, a filamentous eucaryotic green alga, and its associated tailed virus-like particle. Virology. 1980;100:156–65. 18631632 10.1016/0042-6822(80)90561-9

[11] Sicko-Goad L, Walker G. Viroplasm and large virus-like particles in the dinoflagellate *Gymnodiunium uberrimum* . Protoplasma. 1979;99:203–10.

[12] Legendre M, Lartigue A, Bertaux L, Jeudy S, Bartoli J, Lescot M, et al In-depth study of Mollivirus sibericum, a new 30,000-y-old giant virus infecting Acanthamoeba. Proc Natl Acad Sci U S A. 2015;112(38):E5327–35. 10.1073/pnas.1510795112 26351664 PMC4586845

[13] Colson P, Aherfi S, La Scola B, Raoult D. The role of giant viruses of amoebas in humans. Curr Opin Microbiol. 2016;31:199–208. 10.1016/j.mib.2016.04.012 27131020

[14] Moniruzzaman M, Gann ER, LeCleir GR, Kang Y, Gobler CJ, Wilhelm SW. Diversity and dynamics of algal Megaviridae members during a harmful brown tide caused by the pelagophyte, Aureococcus anophagefferens. FEMS Microbiol Ecol. 2016 10.1093/femsec/fiw058 26985013

[15] Netherton CL, Wileman T. Virus factories, double membrane vesicles and viroplasm generated in animal cells. Curr Opin Virol. 2011;1(5):381–7. 10.1016/j.coviro.2011.09.008 . 22440839 PMC7102809

[16] Levasseur A, Bekliz M, Chabrière E, Pontarotti P, La Scola B, Raoult D. MIMIVIRE is a defence system in mimivirus that confers resistance to virophage. Nature. 2016;531(7593):249–52. 10.1038/nature17146 26934229

[17] Boyer M, Azza S, Barrassi L, Klose T, Campocasso A, Pagnier I, et al Mimivirus shows dramatic genome reduction after intraamoebal culture. Proc Natl Acad Sci U S A. 2011;108(25):10296–301. Epub 2011/06/08. 10.1073/pnas.1101118108 ; PubMed Central PMCID: PMCPmc3121840. 21646533 PMC3121840

[18] Elde NC, Child SJ, Eickbush MT, Kitzman JO, Rogers KS, Shendure J, et al Poxviruses deploy genomic accordions to adapt rapidly against host antiviral defenses. Cell. 2012;150:831–41. 10.1016/j.cell.2012.05.049 22901812 PMC3499626

[19] Claverie JM, Abergel C. Giant viruses: the difficule breaking of multiple epistemological barriers. Studies in History and Philosophy of Science Part C: Studies in History and Philosophy of Biological and Biomedical Sciences. 2016; in press. 10.1016/j.shpsc.2016.02.015 26972873

[20] Koonin EV, Starokadomskyy P. Are viruses alive? The replicator paradigm sheds decisive ligh on an old but misguided question. Studies in History and Philosophy of Science Part C: Studies in History and Philosophy of Biological and Biomedical Sciences. 2016;in press. 10.1016/j.shpsc.2016.02.016 PMC5406846 26965225

[21] Satinsky BM, Crump BC, Smith CB, Sharma S, Zielinski BL, Doherty M, et al Microspatial gene expression patterns in the Amazon River Plume. Proc Natl Acad Sci U S A. 2014;111:11085–90. 10.1073/pnas.1402782111. PubMed Central PMCID: PMCPMC4121788. 25024226 PMC4121788

[22] Amin SA, Hmelo LR, van Tol HM, Durham BP, Carloson LT, Heal KR, et al Interaction and signalling between a cosmopolitan phytoplankton and associated bacteria. Nature. 2015;522:98–101. 10.1038/nature14488 26017307

[23] Culley AI, Mueller JA, Belcaid M, Wood-Charlson EM, Poisson G, Steward GF. The characterization of RNA viruses in tropical seawater using targeted PCR and metagenomics. mBio. 2014;5:e01210–14. 10.1128/mBio.01210-14 24939887 PMC4068258

[24] Kopf A, Kostadinova I, Wichels A, Quast C, Glockner FO. Metatranscriptome of marine bacterioplankton during winter time in the North Sea assessed by total RNA sequencing. Marine Genomics. 2015;19:45–6. 10.1016/j.margen.2014.11.001 25479944

